# A Transcriptional Signature of PDGF-DD Activated Natural Killer Cells Predicts More Favorable Prognosis in Low-Grade Glioma

**DOI:** 10.3389/fimmu.2021.668391

**Published:** 2021-09-02

**Authors:** Yuhan Sun, Alexander James Sedgwick, Yaseelan Palarasah, Stefano Mangiola, Alexander David Barrow

**Affiliations:** ^1^Department of Microbiology and Immunology, The University of Melbourne and The Peter Doherty Institute for Infection and Immunity, Melbourne, VIC, Australia; ^2^Institute of Molecular Medicine, University of Southern Denmark, Odense, Denmark; ^3^Division of Bioinformatics, Walter and Eliza Hall Institute, Parkville, VIC, Australia; ^4^Department of Medical Biology, University of Melbourne, Melbourne, VIC, Australia

**Keywords:** NK cell, low grade glioma, NK cell receptor, anti-tumor immunity, The Cancer Genome Atlas

## Abstract

The binding of platelet-derived growth factor D (PDGF-DD) to the NKp44 receptor activates a distinct transcriptional program in primary IL-2 expanded human natural killer (NK) cells. We were interested in knowing if the PDGF-DD-NKp44 pathway of NK cell activation might play a clinically relevant role in anti-tumor immunity. In order to address this question, we determined transcriptional signatures unique to resting, IL-2 expanded, and PDGF-DD activated, NK cells, in addition to different T cell subsets, and established the abundance of these immune cell phenotypes in The Cancer Genome Atlas (TCGA) low-grade glioma (LGG) dataset using CIBERSORT. Our results show that LGG patient tumors enriched for either the PDGF-DD activated NK cell or memory CD8^+^ T cell phenotypes are associated with a more favorable prognosis. Combined cell phenotype analyses revealed that patients with LGG tumors enriched for the PDGF-DD activated NK cell phenotype and the CD4^+^ T helper cell phenotype had a more favorable prognosis. High expression of transcripts encoding members of the killer cell lectin-like receptor (KLR) family, such as KLRK1 and KLRC2, KLRC3 and KLRC4 in LGG tumors were associated with more favorable prognosis, suggesting that these NK cell family receptors may play a prominent role in LGG anti-tumor immunity. Finally, many of the TCGA findings were reciprocated in LGG patients from the Chinese Glioma Genome Atlas (CGGA) dataset. Our results provide transcriptomic evidence that PDGF-DD activated NK cells and KLR family receptors may play an important clinical role in immune surveillance of LGG.

## Introduction

Diffuse and infiltrative low-grade gliomas (LGGs) are derived from the malignant transformation of astrocytes or oligodendrocytes (1). Whilst grade I LGGs are readily resectable benign tumors, grade II LGG display pathologic traits and inexorably progress to high grade gliomas, such as glioblastoma (GBM), with terminal neurological decline (1, 2). Early surgical excision and temozolomide treatment followed by radiotherapy underpin current standards of care but are not curative (3–5). Despite growing understanding of LGG pathogenesis, clinical outcomes have failed to improve particularly for young adults (6). Furthermore, variable rates of progression to lethal disease impede timely clinical intervention and make accurate prognoses difficult (7). Thus, there is an urgent need to understand effective anti-tumor immunity in LGG. Whilst the phenotype and function of tumor-infiltrating lymphocytes (TILs) have been explored for high grade gliomas (8), the prognostic value of TIL subsets and the molecular pathways of tumor recognition for LGG remain unclear.

NK cells preferentially eliminate nascent tumors that have downregulated MHC class-I (MHC-I) (9–11). In homeostasis, NK cells are retained in the tissues surrounding the brain parenchyma by the blood-brain-barrier (BBB) (12, 13). Whilst NK cells have been identified in brain tumors and the surrounding tissue microenvironment (14–16), the mechanisms facilitating NK cell transmigration across the BBB and activation within the brain are poorly defined, although the BBB is more permeable under inflammatory conditions (17–20). Glioma cell lines are readily susceptible to NK cell lysis *in vitro* (21, 22). However, *in vivo* studies reveal a highly vascularized tumor microenvironment that actively subverts immune control (23, 24) and so the significance of NK cell surveillance for gliomas remains to be fully understood (25). Defining immune surveillance mechanisms in those LGG patients with enhanced survival will therefore be critical for the development of novel cancer immunotherapies.

Among others, germline-encoded activating receptors, such as KLRK1 (also known as NKG2D) and the Natural cytotoxicity receptors (NCRs), such as NKp46 (NCR1), NKp44 (NCR2), and NKp30 (NCR3), can synergize to overcome inhibitory thresholds and evoke NK cell anti-tumor functions (26–28). As such, NK cell anti-tumor activity is sensitive to activating receptor surface phenotype and the expression of tumor ligands (29). KLRK1 recognizes a range of ligands upregulated by transformed cells, such as MHC class I chain-related sequence (MIC) A and MICB, which are major determinants of NK cell tumor cytolysis in humans (30–33). Of the NCRs, NKp44 also recognizes a range of cellular and tumor-associated surface ligands, such as Nidogen-1 (34), the heparan sulfate proteoglycan, Syndecan-4 (35), a subset of HLA-DP molecules (36), a splice variant of the mixed lineage leukemia 5 (MLL5) gene (37), and proliferating cell nuclear antigen (PCNA) (38), that have all been reported to positively or negatively regulate NK cell function (39, 40). Recently, PDGF-DD was shown to induce signaling from the activating NKp44 immunoreceptor (41).

The platelet-derived growth factor (PDGF) family are comprised of four polypeptides that assemble into five dimeric isoforms, PDGF-AA, PDGF-BB, PDGF-AB, PDGF-CC, and PDGF-DD. PDGFs play essential roles in embryonic development, cell proliferation, cell migration, survival and chemotaxis by engaging PDGF receptors (PDGFRs) that are mostly expressed by mesenchymal cells (42). PDGF-DD is a potent mitogen that plays an important role in wound healing and blood vessel maturation during angiogenesis by inducing PDGFR-β signaling on mesenchymal cells (43, 44). In brain cancer, PDGF-DD binding to PDGFR-β can induce pro-tumorigenic signaling that drives glioma progression (45–49).

PDGF-DD stimulation of NKp44 induced NK cell secretion of IFN-γ and TNF that arrest tumor cell proliferation *in vitro* and may confer a survival benefit in GBM (41). In support of this, PDGF-DD is abundantly expressed in GBM, suggesting a novel mode of NK cell tumor surveillance (50), but the clinical significance of the NKp44-PDGF-DD pathway for anti-tumor immunity in many other human cancers including LGG remains unclear.

Here, we employed a computational approach to investigate the clinical impact of the relative enrichment of resting, IL-2 expanded, and PDGF-DD activated NK cell phenotypes in the LGG tumor microenvironment. To achieve this, we used transcriptional signatures from the three NK cell activation states and estimated their relative abundance in LGG tumor specimens from TCGA database and tested the association with curated progression-free survival (51).

## Methods

### Material Availability

The R codes for the analyses presented in this study are available at RAGG3D/LGG_SPANK (github.com). An overview of the methods used in this study are shown in **Supplementary Figure 1**.

### Data Collection

Gene transcript-abundance and patient clinical information were collected from TCGA through the GDC Data Portal (52) and the CGGA (53–56). Progression-free survival information was used as a measure of clinical outcome (51). The cell-type specific transcriptional signatures were derived from a large collection of RNA sequencing samples spanning a wide range of cell types. For NK cells, experimentally derived dataset for IL-2 expanded (27 biological replicates), PDGF-DD activated *via* NKp44 signaling (4 biological replicates), and resting (25 biological replicates from 6 datasets) (41) were included. For other cell types, the data collected was from the following datasets: BLUEPRINT (57), Monaco et al. (58), ENCODE (59), Squires et al. (60), GSE77808 (61), Tong et al. (62), PRJNA339309 (63), GSE122325 (64), FANTOM5 (65), GSE125887 (66), GSE130379 (67), GSE130286 (68).

### Transcriptional Signatures

In order to derive transcriptional signatures of 21 cell types (memory B cell, naive B cell, immature dendritic myeloid cell, immature dendritic myeloid cell, endothelial, eosinophil, epithelial, fibroblast, macrophage M1 and M2, mast cell, monocyte, neutrophil, ReNK, IL2NK, SPANK, memory CD4 T cell, memory CD8 T cell, naive CD8 T cell, gamma-delta T cell and helper T cell), a total of 592 highly curated (i.e. for which identity was confirmed in the literature), non-redundant biological replicates (including 25 ReNK samples, 27 IL2NK samples and 4 SPANK samples), have been used. Due to the sparse nature of heterogeneous data sets, the expected value and variability of gene transcription abundance was inferred for each cell type using a publicly available Bayesian statistical model (github: stemangiola/cellsig), based on a negative binomial data distribution (69). This model allows to fit incomplete data (e.g. transcript abundance of one gene for which data is available in a subset of reference biological replicates) and calculate theoretical data distributions of cell-type/gene pairs. The cell-type transcriptional marker selection was based on the pairwise comparison of each cell type within cell-type categories along a cell differentiation hierarchy (**Supplementary Figure 2**) (70). For example, all cell-type permutations from the root node of level one (including epithelial, endothelial, fibroblasts and immune cells) were interrogated in order to select the genes for which the theoretical transcript abundance distribution (data generated from the posterior distribution) was higher for one cell type compared to another. This was executed calculating the distance of the upper and lower 95% credible intervals, respectively (obtained from cellsig). From each comparison, the top 5, 10 and 20 genes per cell-type pair were selected from levels 1, 2, and 3 (**Supplementary Figure 2**), and the union of all genes was taken as overall marker gene list. This hierarchical approach favors the identification of marker genes that distinguish broad cell-type categories as well as specific activation phenotypes.

### Estimation of the Association of Cell-Type Abundance With Relapse-Free Patient Survival

In order to estimate the cell type relative abundance for each biological replicate, we used the algorithm CIBERSORT (71) with our RNA sequencing-derived gene marker signature. In order to estimate the clinical relevance of NK activation phenotypes (72) (73),for each cancer-type/cell-type pair, Kaplan-Meier (KM) survival curves (74) were calculated from the median split CIBERSORT-inferred proportions through the R framework tidybulk. Percent survival vs time-to-event statistics were calculated by the Log-rank (Mantel-Cox) Test (75). Statistics of KM curves were performed by log-rank test then adjusted by the Benjamini-Hochberg (BH) procedure. A table of all p-values prior to adjustment is provided in **Supplementary Table 1**.

Data analysis and visualization were performed using the R environment in RStudio (76). Packages include tidyverse (77), tidybulk (73), tidyHeatmap (78), survminer (79), survival (80, 81), foreach (82), org.Hs.eg.db (83), cowplot (77), ggsci (84), GGally (85), gridExtra (86), grid (76), reshape (87), Hmisc (88), and viridis (89).

### Benchmark of the Transcriptional Signatures

In order to visually evaluate the ability of the marker gene selection in segregating cell types, we first performed principal component analyses (PCA) (90) for three levels of cell differentiation: (i) the NK activated states, (ii) all NK cells, and (iii) all cell types. Briefly, the raw read counts were normalized by trimmed mean of M values (TMM) using tidybulk function *scale_abundance*, whilst CGGA transcripts were already normalized by transcripts per kilobase of exon model per million mapped reads (TPM) *via* RNA-seq by expectation Maximization (RSEM). PCA analyses were performed by *reduce_dimensions(method=“PCA”)* (73). To directly test whether the selected signature for PDGF-DD activated NK cells was suitable to accurately detect changes in cell abundance across samples with a censored time-to-event, we implemented a test on simulated data. First, for a selected number of patients N, we sampled the progression-free survival time from the clinical annotation of the LGG patient cohort. Cell type proportions were simulated using a Dirichlet distribution, according to a linear model with a slope value S and a progression-free survival as factor of interest. For each simulated dataset, the slope S was assigned to only one cell type, and the slope of 0 to all the others (i.e. only one cell type changing for each simulated tissue mixture). The intercept (baseline proportion) was defined to be the same for all cell types. The simulated proportions were used to compose the in-silico mixtures. For each simulated dataset, the transcriptional profile of each cell type was sampled at random from the reference dataset. In order to test the accuracy of our method against the presence of foreign cell types (of which transcriptomic signature was not included in our reference set), a proportion P of neural cells was added to the mixture. The framework tidybulk was used to infer the cell type proportions through CIBERSORT and perform a multiple cox regression on the predicted proportions (logit-transformed) (81), with progression-free survival censored time as a covariate. That is, for half of the N samples, the survival time was censored to half of its value. The significance calls were compared with the ground truth to generate a receiver operating characteristic (ROC) curve. For each simulation condition (values of N, S, and P), 63 test runs were performed with one variable cell type each. A range of simulation conditions were tested, ranging N from 250 to 1000, S from 0.2 to 1, and P from 0 to 0.8.

## Results

### NK Cell Phenotypes Have Unique Transcriptional Profiles

Given the innate ability of NK cells to lyse tumor cells and secrete potent anti-tumor cytokines, such as IFN-γ and TNF, prior to immunization, we hypothesized that NK cells of unique phenotype may infiltrate different cancers and confer anti-tumor immunity. Specifically, we were motivated by the recent discovery of PDGF-DD as a ligand for the activating NK cell receptor NKp44 (41) and whether this mechanism of NK cell stimulation might constitute a clinically relevant pathway of anti-tumor immunity. In order to answer this question, we gathered publicly available RNA sequencing data from 21 different immune and stromal cell types (see Methods) in order to define marker genes that may distinguish resting (91), IL-2 expanded, and PDGF-DD activated, NK cell phenotypes (41).

**Figure 1:**
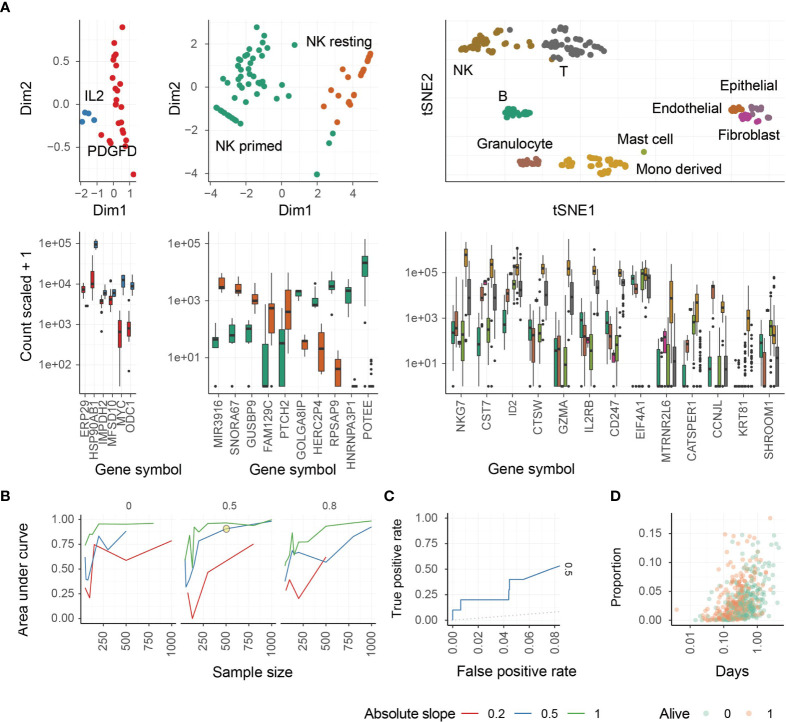
Identification of different NK cell phenotypes using transcriptional signatures. **(A)** Study of transcriptomic signatures for activated NK cells (left), all NK cell phenotypes (middle), and all cell types (right). On the top the principal components or the t-distributed stochastic neighbor embedding dimensions for the biological replicate within the reference dataset are shown. On the bottom, the relative transcriptional abundance is shown for marker genes. **(B)** Test for accuracy of the inference of PDGF-DD activated NK cell proportion from simulated mixtures. This plot represents the accuracy of the combination of CIBERSORT with Cox-regression by inferring associations between convoluted tissue composition and survival time. The three facets represent low-to-high proportion of missing information (proportion of total cells being of neural origin in the simulated mixtures, for which signature was not present in the reference). Data was simulated across a range of sample-size and slopes. A simulation condition that represents the associations we detected in the TCGA database is circled. **(C)** Receiver Operating Characteristic (ROC) curve, measuring the accuracy (true-positive and false-positive) for the simulated mixture circled in panel **(C, D)** The underlying association between the positively associated cell types with survival days, of the simulated dataset circled in panel **(C)**.

We next performed a principal component analysis (PCA) to determine the ability of these marker genes to segregate all three NK cell phenotypes from each other and from other cell types, such as T cells (**Figure 1A**). NK cell activation states are associated with the first principal components (**Figure 1A**, left and middle panels), and NK cells overall are defined by a unique cluster when compared with other major cell types (**Figure 1A**, right panel). NK cell phenotypes segregated from other cell types and from each other by PCA.

We next performed a benchmark for the inference of changes in the relative abundance of PDGF-DD activated NK cells in association with survival information for artificial tissue mixtures built from our reference data set (see Methods). This benchmark measured the ability of the PDGF-DD activated NK cell signature to extract clinically-relevant information from TCGA whole tissue RNA sequencing data (**Figure 1B**). The benchmark showed a high accuracy (area under curve) across simulation settings including magnitude of variability, sample-size, and proportion of unknown cells (please see Methods). An accuracy of 0.75 (representing the area under the ROC curve) was reached for simulation settings that match our findings on TCGA data (slope and sample size; **Figures 1C, D**). We refer to the different NK cell phenotypes as: resting NK cells (ReNK), IL-2 expanded NK cells (IL2NK), and the signature of PDGF-DD activated NK cells (SPANK), respectively, and the transcript abundance of each marker gene in these NK cell phenotypes are shown in **Supplementary Table 2**.

### The SPANK Is Associated With Improved Prognosis in TCGA LGG Dataset

**Figure 2:**
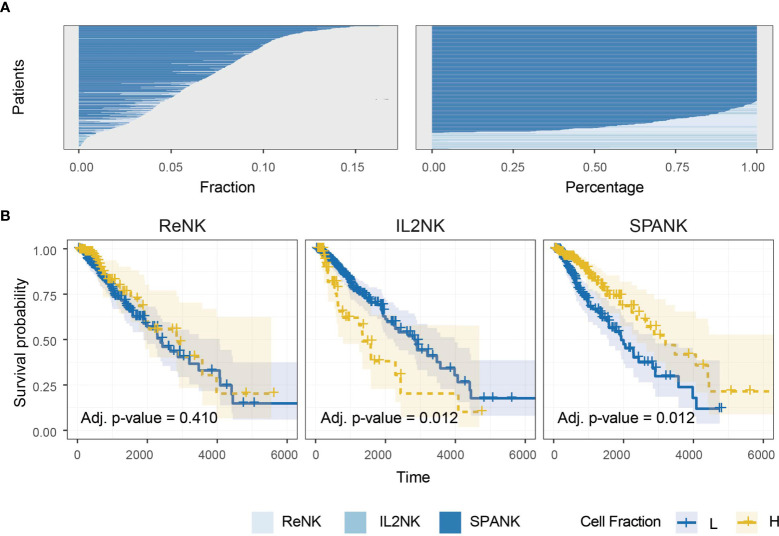
Abundance of NK cell phenotypes and association with survival in the TCGA LGG dataset. **(A)** Abundance of NK cell phenotypes (fraction and percentage) for TCGA LGG cohort, the SPANK is the most abundant NK cell phenotype in LGG. **(B)** KM curves for all three NK cell phenotypes for TCGA-LGG; high tumor abundance of SPANK is associated with a beneficial LGG patient outcome compared to ReNK and IL2NK.

Previous studies have implicated NK cells in immune responses to glioma (15, 92–95). We next sought the association of the ReNK, IL2NK, and SPANK phenotypes with LGG. The SPANK was more abundant than either the ReNK or IL2NK phenotypes in LGG tumors from TCGA (**Figure 2A**). LGG tumors enriched for the SPANK were associated with greater overall patient survival compared to the ReNK or IL2NK phenotypes (**Figure 2B**). These results show that tumor abundance of a distinct NK cell phenotype is associated with cancer patient survival, such as LGG. Moreover, these data also suggest that LGG tumors express PDGF-DD which may activate pro-tumorigenic pathways.

### PDGFD Expression Is Associated With LGG Invasion and Poor Prognosis

**Figure 3:**
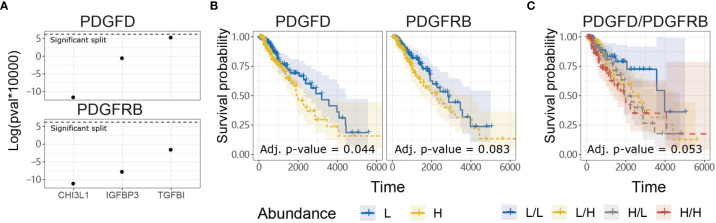
*PDGFD* expression is associated with tumor invasion and poor prognosis compared to *PDGFRB* in TCGA LGG dataset. **(A)** Tumor expression of *PDGFD* and *PDGFRB* were positively correlated with the expression of *TGFBI, IGFBP3, and CHI3L1* which play important roles in glioma invasion and migration. **(B)** KM survival curves constructed for *PDGFD* or *PDGFRB* expression in LGG tumors. **(C)** KM curves constructed for combinations of *PDGFD* and *PDGFRB* abundance in LGG tumors.

In contrast to evoking NK cell anti-tumor functions through PDGF-DD binding to NKp44 (41), PDGF-DD (encoded by *PDGFD*) binding to PDGFR-β (encoded by *PDGFRB*) induces pro-tumorigenic signaling pathways that are detrimental for cancer patient survival (42, 47, 96, 97). Expression of a three-gene signature, comprised of *TGFBI*, *IGFBP3*, and *CHI3L1*, has previously been associated with glioma tumor cell invasion and migration and poor patient survival (98). Tumor expression of *TGFBI*, *IGFBP3*, and *CHI3L1* were positively correlated with *PDGFD* and *PDGFRB* expression in TCGA LGG dataset, respectively (**Figure 3A**) (98).

Since *PDGFD* and *PDGFRB* were associated with genes involved in glioma tumor cell invasion and migration, we next examined the relationship between tumor expression of *PDGFD* or *PDGFRB* and LGG patient survival. LGG patients with low tumor expression of *PDGFD* had more favorable prognosis compared to LGG patients with high tumor expression of *PDGFD* (**Figure 3B**). Higher LGG tumor expression levels of *PDGFRB* alone displayed a trend towards poor survival (**Figure 3B**) when *PDGFD* expression was low, but this was not statistically significant (**Figure 3C**). These data show that high tumor expression of *PDGFD* is primarily associated with poor prognosis compared to *PDGFRB* expression in TCGA LGG dataset.

### SPANK Abundance Mitigates the Pro-Tumorigenic Effects of PDGFD in TCGA LGG

**Figure 4:**
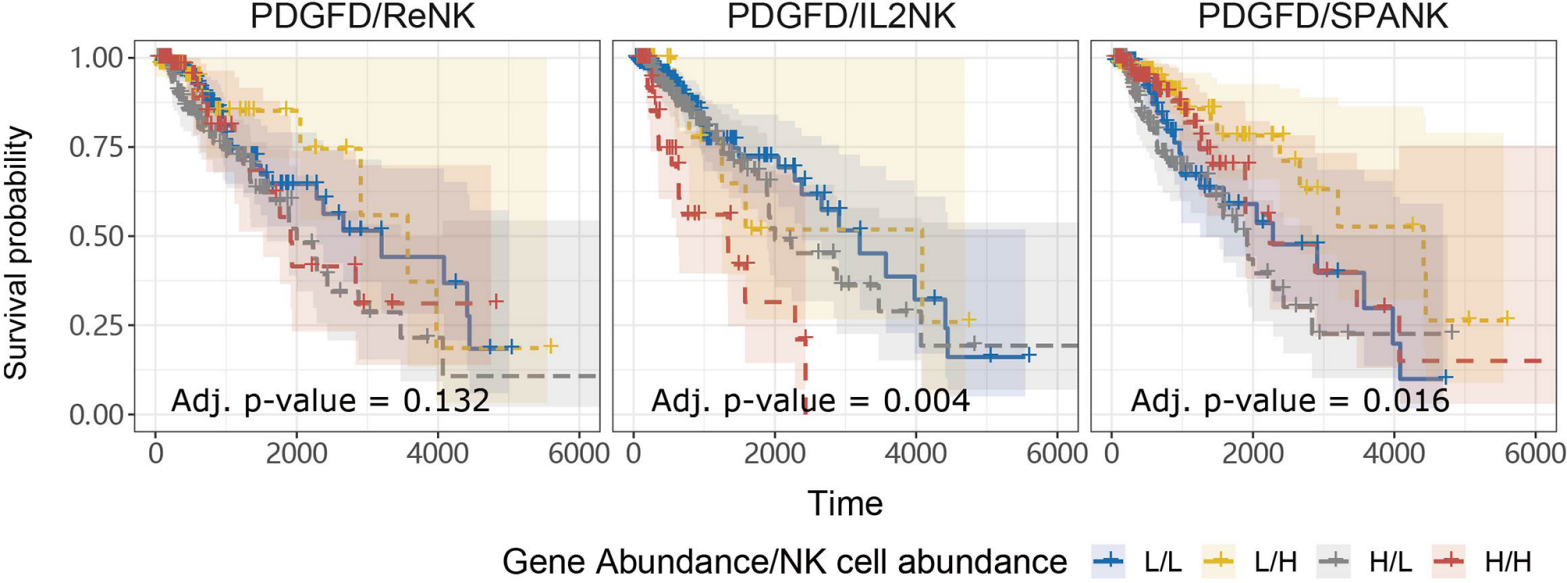
SPANK abundance alleviates the pro-tumorigenic effects of *PDGFD* on TCGA LGG prognosis. Combined LGG patient survival analysis stratified for tumor expression of *PDGFD* and each NK cell phenotype, ReNK, IL2NK, and SPANK, respectively. KM curves display LGG patient survival plotted in all four combinations for each stratum, respectively (L/L, L/H, H/L, and H/H). For LGG tumors with either high or low *PDGFD* expression, a high tumor abundance of SPANK is associated with improved LGG prognosis.

Anti-tumor immunity would be expected to curtail pro-tumorigenic factors and benefit patient survival. In addition to pro-tumor functions, we hypothesized that tumors enriched for the SPANK would contribute to anti-tumor immunity resulting in a more favorable TCGA LGG prognosis (41). In order to assess whether the abundance of these NK cell phenotypes counteracted the pro-tumorigenic expression of *PDGFD* and thus improve TCGA LGG prognosis, we next determined the progression-free survival of LGG patients with tumors stratified for *PDGFD* expression and abundance of either the ReNK, IL2NK, or SPANK phenotypes. When LGG tumors were stratified for PDGFD expression, patients with tumors enriched for the SPANK had a more favorable prognosis compared to LGG patients with a lower tumor abundance of SPANK (e.g. compare H^PDGFD^/H^SPANK^, red KM curve, to H^PDGFD^/L^SPANK^, grey KM curve and compare L^PDGFD^/H^SPANK^, yellow curve, to L^PDGFD^/L^SPANK^, blue curve) (**Figure 4**). In contrast, this was not observed for either ReNK or IL2NK (**Figure 4**). These results show that LGG tumors enriched for the SPANK may mitigate the detrimental effect of *PDGFD* expression on the prognosis of TCGA LGG patient cohort compared to the ReNK or IL2NK phenotypes.

### Memory CD8^+^ T Cell Abundance Mitigates the Pro-Tumorigenic Effects of PDGFD in TCGA LGG

**Figure 5:**
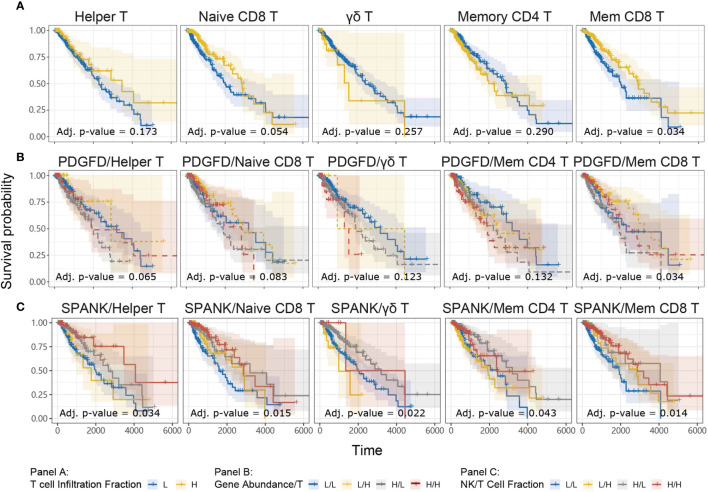
Tumor abundance of memory CD8^+^ and Helper T cells influences TCGA LGG prognosis KM curves displaying the survival of LGG patients split by median fraction into low (L) and high (H) tumor expression of **(A)** each T cell phenotype alone, **(B)** *PDGFD* and each T cell phenotype, respectively, or **(C)** each NK cell and each T cell phenotype, respectively. KM curves display the survival of LGG patients plotted in all combinations for each stratum.

For a given cancer, it is likely that immune subsets other than NK cells infiltrate the tumor microenvironment to elicit anti-tumor immunity, particularly T cells. We were interested in knowing whether the abundance of a given T cell subset in the LGG tumor microenvironment is associated with anti-tumor immunity. TCGA LGG tumors enriched for the memory CD8^+^ T cell phenotype were associated with improved prognosis, but not the naïve, γδ, CD4^+^ memory, or Helper, T cell phenotypes (**Figure 5A**). Our analyses show that TCGA LGG patients enriched for the memory CD8^+^ T cell phenotype have improved prognosis.

In order to determine whether each T cell phenotype might counteract the detrimental expression of *PDGFD* on LGG prognosis, similarly to the SPANK, we next determined progression-free survival for LGG patients with tumors stratified for expression of *PDGFD* and the abundance of each T cell phenotype, respectively (**Figure 5B**). Using this approach, LGG tumors enriched for the memory CD8^+^ T cell phenotype were associated with improved prognosis (**Figure 5B**). These results show that LGG tumors enriched for the memory CD8^+^ T cell phenotype mitigate the pro-tumorigenic effects of *PDGFD* because they are associated with improved prognosis.

### The SPANK and Helper T cell Phenotype Are Associated With Improved LGG Prognosis

Given that the abundance of NK and T cell phenotypes differ markedly in LGG tumors (**Supplementary Figure 3**), we were interested in understanding the relative contribution of SPANK and T cell phenotypes for LGG prognosis, respectively. We therefore determined patient survival for TCGA LGG tumors stratified for the abundance of SPANK and each respective T cell subset (**Figure 5C**). Interestingly, LGG tumors enriched for the SPANK and CD4^+^ T helper phenotypes (TH) had improved prognosis compared to other strata e.g. compare H^SPANK^/H^TH^ to either H^SPANK^/L^TH^ or L^SPANK^/H^TH^ or L^SPANK^/L^TH^ (**Figure 5C**, column 1). In contrast, LGG tumors enriched for the SPANK and memory CD8^+^ T cell phenotypes (CD8mem) did not further improve LGG patient survival compared to other strata e.g. compare H^SPANK^/H^CD8mem^ to either L^SPANK^/H^CD8mem^ or H^SPANK^/L^CD8mem^ (**Figure 5C**, column 5). We conclude that LGG tumors enriched for the SPANK and CD4^+^ T helper cell phenotypes are associated with improved LGG prognosis in TCGA. These results provide new insights into the possible cooperation between different NK and T cell subsets for LGG anti-tumor immunity which may inform adoptive cell therapies.

### Critical Role for Killer Cell Lectin-Like Receptor Family Members in LGG Anti-Tumor Immunity

**Figure 6:**
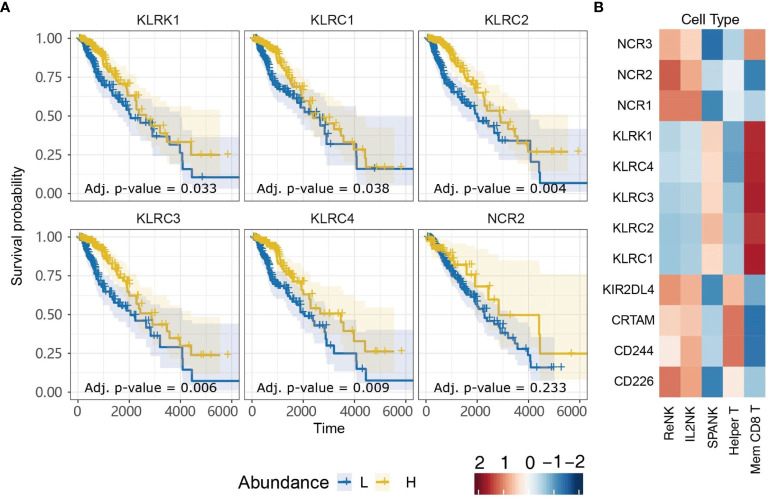
Killer cell Lectin-like Receptor family expression is associated with more favorable TCGA LGG prognosis. **(A)** KM plots displaying progression-free survival of LGG patients split by median fraction into low (L) and high (H) tumor expression for the NK cell receptor genes: *KLRK1, KLRC1, KLRC2, KLRC3, KLRC4*, or *NCR2.* **(B)** Heatmap displaying correlations between expression of each NK cell receptor transcript (y-axis) and each NK cell and T cell phenotype (x-axis), respectively.

NK cells express a family of germline-encoded activating and inhibitory surface receptors that engage in cancer immune surveillance, which can also be expressed by memory CD8^+^ T cells. However, the NK cell family receptors most critical for anti-tumor immunity in LGG remain unclear. Given that LGG patients in TCGA with tumors enriched for the SPANK or memory CD8^+^ T cells were associated with improved prognosis, we were interested in analyzing whether tumor expression of transcripts encoding NK cell family receptors was also associated with improved prognosis for TCGA LGG patients. LGG tumors with high expression of the *KLRK1*, *KLRC1*, *KLRC2*, *KLRC3*, or *KLRC4* transcripts encoding the NKG2D, NKG2A, NKG2C, NKG2E, and NKG2F NK cell receptors, respectively, were associated with improved prognosis (**Figure 6A**). In contrast, high LGG tumor expression of *CD226*, *CD244*, *CRTAM*, *KIR2DL4*, *NCR1*, or *NCR3* encoding the DNAM-1, 2B4, CRTAM, NKp46 and NKp30 NK cell receptors, respectively, were not associated with prognosis (**Supplementary Figure 4**). Moreover, expression of the *KLRK1*, *KLRC1*, *KLRC2*, *KLRC3*, and *KLRC4* receptor genes were overwhelmingly positively correlated with the SPANK and memory CD8^+^ T cell phenotypes in TCGA LGG tumors (**Figure 6B**). These results show that high expression of the Killer cell lectin-like receptor (KLR) family in LGG tumors is associated with improved prognosis, suggesting that expression of KLR receptors may be critical for regulating anti-tumor immunity in LGG.

### The SPANK and Memory CD8^+^ T Cell Phenotypes Are Associated With KLRC2 Expression and More Favorable Prognosis in CGGA LGG Patients

**Figure 7:**
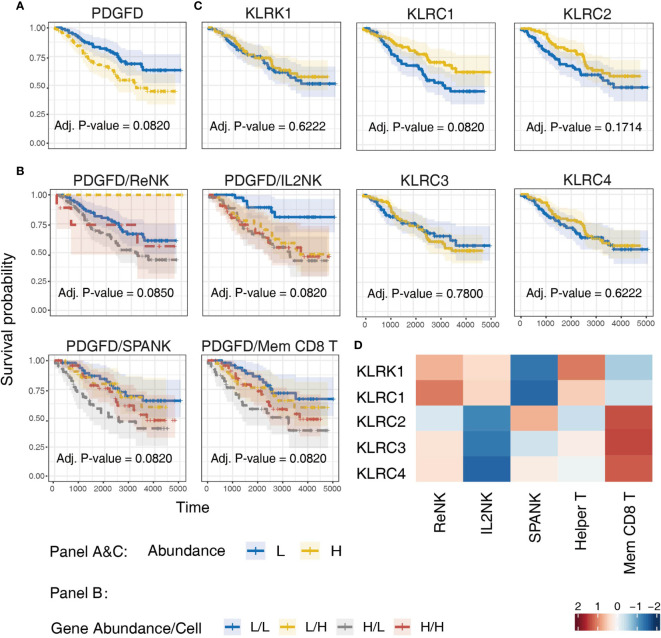
Validation of TCGA findings using the CGGA LGG dataset. KM curves displaying the survival of CGGA LGG patients split by median fraction into low (L) and high (H) tumor expression of: **(A)** *PDGFD*, **(B)** *PDGFD* and either NK cell or memory CD8^+^ T cell phenotypes, respectively, and **(C)** NK cell receptor genes, *KLRK1, KLRC1, KLRC2, KLRC3*, or *KLRC4*, respectively. KM curves display the survival of LGG patients plotted in all combinations for each stratum. **(D)** Heatmap displaying correlations between the expression of each NK cell receptor transcript (y-axis) and NK cell and T cell phenotypes (x-axis) in CGGA LGG tumors, respectively.

Since LGG tumors enriched for expression of the SPANK and KLR family receptors were associated with improved prognosis in the TCGA LGG patient cohort, we next sought to validate these findings using another glioma patient dataset, such as the CGGA (**Figure 7**). Similar to TCGA LGG patient cohort, high tumor expression of *PDGFD* in CGGA LGG patients was associated with a poor prognosis (**Figure 7A**). Moreover, LGG tumors enriched for the SPANK and memory CD8^+^ T cell phenotypes were associated with improved prognosis when CGGA tumors were also stratified for *PDGFD* expression compared to the IL2NK phenotype (**Figure 7B**). In contrast to TCGA LGG dataset, high tumor expression of *KLRC1* and *KLRC2* in CGGA LGG patients were associated with improved prognosis, but not *KLRC3*, *KLRC4* or *KLRK1* (**Figure 7C**). Like TCGA, high expression of *KLRC2*, which encodes the activating NKG2C receptor, was also associated with the SPANK and memory CD8^+^ T cell phenotypes in LGG tumors (**Figure 7D**). However, in contrast to TCGA, expression of *KLRC1*, which encodes the inhibitory NKG2A receptor, was associated with the ReNK phenotype and not the SPANK or memory CD8^+^ T cell phenotypes in CGGA LGG tumors (**Figure 7D**). Similar to TCGA, these results show that high tumor expression of *PDGFD* is associated with poor CGGA LGG prognosis, and tumors enriched for the SPANK and memory CD8^+^ T cell phenotypes have improved prognosis. Moreover, like TCGA, high LGG tumor expression of *KLRC2* is also associated with the SPANK and memory CD8^+^ T cell phenotypes and improved prognosis of CGGA LGG patients.

## Discussion

The clinical relevance of NK cells in cancer immune surveillance, particularly for solid tumors, remains unclear. We hypothesized that differential tumor enrichment of NK cells in different activation states may contribute to anti-tumor immunity. To investigate this important question, we determined TS from experimental RNA-seq datasets derived from NK cells in three different functional states; ReNK, IL2NK, and SPANK. Using this unbiased approach, we found that enrichment of the SPANK phenotype in LGG tumors was associated with improved prognosis in TCGA and CGGA datasets. The SPANK was derived from RNA-seq data from NK cells that had been stimulated with PDGF-DD, suggesting that PDGF-DD is expressed in the LGG tumor microenvironment.

In addition to activating NK cells, PDGF-DD binding to PDGFR-β can induce pro-tumorigenic signaling pathways. We reasoned that high expression of the genes for PDGF-D and PDGFR-β in LGG might predict poor LGG cancer prognosis (42, 47, 96, 97). In support of this, *PDGFD* and *PDGFRB* were positively correlated with a three-gene signature (*TGFBI*, *IGFBP3*, and *CHI3L1*) associated with glioma tumor cell invasion and migration and poor patient survival (98). Indeed, our analysis shows that LGG patients with high tumor expression of *PDGFD* had a poor prognosis in both TCGA and CGGA cohorts (42, 47, 96, 97). This model awaits confirmation in mouse models of glioma to determine whether PDGF-DD/PDGFR-β signaling can be targeted to restrict glioma tumor cell migration and invasion or even progression to higher glioma grades.

Given that the expression of *PDGFD* was primarily associated with pro-tumor pathways and poor prognosis, we further hypothesized that enrichment of the SPANK phenotype in LGG tumors may be associated with effective anti-tumor immunity and improved prognosis. High tumor abundance of the SPANK was associated with improved prognosis when LGG tumors were stratified for the expression of *PDGFD* in both TCGA and CGGA patient cohorts. Our data show that the relative abundance of the SPANK may counteract the pro-tumorigenic properties of *PDGFD* expression and improve LGG prognosis. Interestingly, Nidogen-1 (34), the heparan sulfate proteoglycan, Syndecan-4 (35), a subset of HLA-DP molecules (36), a splice variant of the MLL5 gene (37), and PCNA (38), have all been reported to bind and regulate NKp44 signaling, and it will be interesting to determine whether expression of these latter genes in the LGG tumor microenvironment can influence the association between the NK cell phenotypes that we describe and LGG prognosis.

It is very likely that NK cells are not the sole mediators of anti-tumor immunity *in vivo* and our analyses revealed that enrichment of the CD8^+^ memory T cell phenotype in LGG tumors was also associated with improved prognosis when LGG tumors were stratified for high or low tumor expression of *PDGFD* in both TCGA and CGGA patient cohorts. Interestingly, stratifying LGG tumors for T cell subsets and NK cells phenotypes revealed new insights into potential cooperation between these innate and adaptive immune cell subsets that was not revealed from the analysis of these immune cell phenotypes alone. For example, LGG patients with tumors enriched for the SPANK and CD4^+^ T helper phenotypes had improved survival suggesting that adoptive transfer of NK cells with CD4^+^ T helper cells may represent a novel therapeutic approach for LGG. Again, these computational results await confirmation in pre-clinical mouse models of glioma to determine whether the adoptive transfer of NK cells with CD4^+^ T helper cells can restrict glioma tumor cell migration and invasion or possibly even progression to higher glioma grades.

Finally, high tumor expression of the NK cell receptor genes; *KLRK1*, *KLRC1*, *KLRC2*, *KLRC3*, and *KLRC4* that encode the NKG2D, NKG2A, NKG2C, NKG2E and NKG2F, respectively, were all associated with improved prognosis and positively correlated with the SPANK and memory CD8^+^ T cell phenotypes, suggesting expression of these KLR NK cell receptor family gene products are important for LGG anti-tumor immunity in TCGA patient cohort.

Interestingly, the KLRC1 receptor, also known as NKG2A, is expressed as a heterodimer with CD94 on the surface of NK cells and T cells (99). CD94/NKG2A can bind to HLA-E as ligand to negatively regulate signaling from other activating KLR family members including KLRC2, known as NKG2C, which also heterodimerizes with CD94 to bind HLA-E (100, 101). Interestingly, KLRC1 has recently been shown to function as a checkpoint inhibitor that when blocked can promote NK cell and CD8^+^ T cell-mediated anti-tumor immunity (102, 103). Since NK cell effector function is regulated by the balance of signaling from an array of germline-encoded activating and inhibitory receptors (29, 104), it is possible that signaling from the activating KLRK1 (NKG2D), NCR2 (NKp44) and KLRC2 (NKG2C) receptors, which are all associated with improved survival (**Figure 6A**), may cooperate to overcome any inhibitory threshold set by KLRC1 (NKG2A) in TCGA LGG patient cohort (103).

High tumor expression of *KLRC1* and *KLRC2* were also associated with improved survival in the CGGA LGG patient cohort, but not KLRK1, KLRC2 or KLRC4. However, the SPANK and CD8^+^ T cell phenotypes were associated with expression of *KLRC2* and not *KLRC1* in the CGGA LGG patients, suggesting the balance of signaling may favor KLRC2 activation in the CGGA LGG patient cohort compared to TCGA LGG cohort. Given our results and those from other laboratories, it will be interesting to determine the expression of HLA-E in LGG and KLR family receptors on glioma-infiltrating NK cells and CD8^+^ T cells in different ethnic groups and to test whether blocking the inhibitory function of KLRC1 can enhance the anti-tumor activity of NK cells and CD8^+^ T cells in LGG and other brain cancers (102, 103). Finally, using CIBERSORT, we have uncovered an intriguing association between tumor expression of *PDGFD* and tumor enrichment of the SPANK and T helper and memory CD8^+^ T cell signatures, that may be important for LGG patient survival. However, it is premature to conclude that the SPANK or the T cell signatures play a definitive role in LGG survival and future studies will aim to determine the biological significance of the SPANK and different T cell phenotypes and NK cell receptors, such as the KLR family, in LGG patient survival.

## Data Availability Statement

The original contributions presented in the study are included in the article/**Supplementary Material**. Further inquiries can be directed to the corresponding authors.

## Author Contributions

AB, SM, YS, and YP contributed to the conception and design of the study. YS and SM performed computational and statistical analysis. AB and YS wrote the first draft of the manuscript. SM and AS wrote sections of the manuscript. AB and SM contributed equally and are joint senior authors. All authors contributed to the article and approved the submitted version.

## Funding

This work was funded by a MRFF research acceleration grant APP1162217 awarded to AB, and a University of Melbourne PhD scholarship awarded to YS. SM is funded by a Galli Early Career Research Fellowship.

## Conflict of Interest

The authors declare that the research was conducted in the absence of any commercial or financial relationships that could be construed as a potential conflict of interest.

## Publisher’s Note

All claims expressed in this article are solely those of the authors and do not necessarily represent those of their affiliated organizations, or those of the publisher, the editors and the reviewers. Any product that may be evaluated in this article, or claim that may be made by its manufacturer, is not guaranteed or endorsed by the publisher.

## Supplementary Material

Supplementary Figure 1 Computational pipeline. In addition to the three NK cell functional phenotypes, bulk RNA-seq data was collected from open online resources for 21 immune and stromal cell types including five T cell subsets, two B cell subsets, two dendritic cells subsets, two macrophage subsets, eosinophils, mast cells and neutrophils, fibroblasts, endothelial and epithelial cells. Secondly, we selected marker genes by pairwise comparison and CIBERSORT to create our transcriptional signature (TS). Thirdly, we input our TS and RNA-seq matrix of TCGA cancer patients into CIBERSORT to estimate the proportion of each cell type in each patient. Finally, we performed a series of statistical analysis using the immune cell type profiles and clinical results of all TCGA cancer patients.

Supplementary Figure 2 The Cell differentiation hierarchy used in marker gene selection. The higher levels are differentiated from cells in the lower levels (L1 differentiates into L2, L2 differentiates into L3, and L3 differentiates into L4).

Supplementary Figure 3 Profiles of NK and T cell subset phenotypes in LGG patients. **(A)** Profiles showing original fractions of NK and T cell phenotypes in LGG tumors **(B)** Proportions of total NK and T cell abundance in LGG tumors.

Supplementary Figure 4 Kaplan-Meier curves of NK cell receptor family genes in LGG patients. Survival analysis of receptor genes *CD226* (encoding DNAM-1), *CD244* (encoding 2B4/SLAMF4)*, CRTAM, KIR2DL4, NCR1* (encoding NKp46) and *NCR3* (encoding NKp30) in TCGA-LGG. High expression of CD226 gene is significantly related to poor survival. Low (L) and high (H) expression groups were split by median TMM normalized counts. Statistics was performed by log-rank test.

Supplementary Table 1 Log rank p values and Benjamini-Hochberg corrected p values. Log rank p-values of Kaplan-Meier curves were all corrected by BH procedure (/ = unchanged).

Supplementary Table 2 NK phenotype TS transcripts. The abundance of each marker gene in the TS of NK cell phenotypes.
