# Supplementary material for: A Transcriptional Signature of PDGF-DD Activated Natural Killer Cells Predicts More Favorable Prognosis in Low-Grade Glioma
Source: Front Immunol. 2021 Sep 2;12:668391. doi: 10.3389/fimmu.2021.668391 (PMC8444979; doi:10.3389/fimmu.2021.668391)
Supplement: Supplementary Figure 1 — Computational pipeline. In addition to the three NK cell functional phenotypes, bulk RNA-seq data was collected from open online resources for 21 immune and stromal cell types including five T cell subsets, two B cell subsets, two dendritic cells subsets, two macrophage subsets, eosinophils, mast cells and neutrophils, fibroblasts, endothelial and epithelial cells. Secondly, we selected marker genes by pairwise comparison and CIBERSORT to create our transcriptional signature (TS). Thirdly, we input our TS and RNA-seq matrix of TCGA cancer patients into CIBERSORT to estimate the proportion of each cell type in each patient. Finally, we performed a series of statistical analysis using the immune cell type profiles and clinical results of all TCGA cancer patients. [file Presentation_1.ppt]

## Slide 1
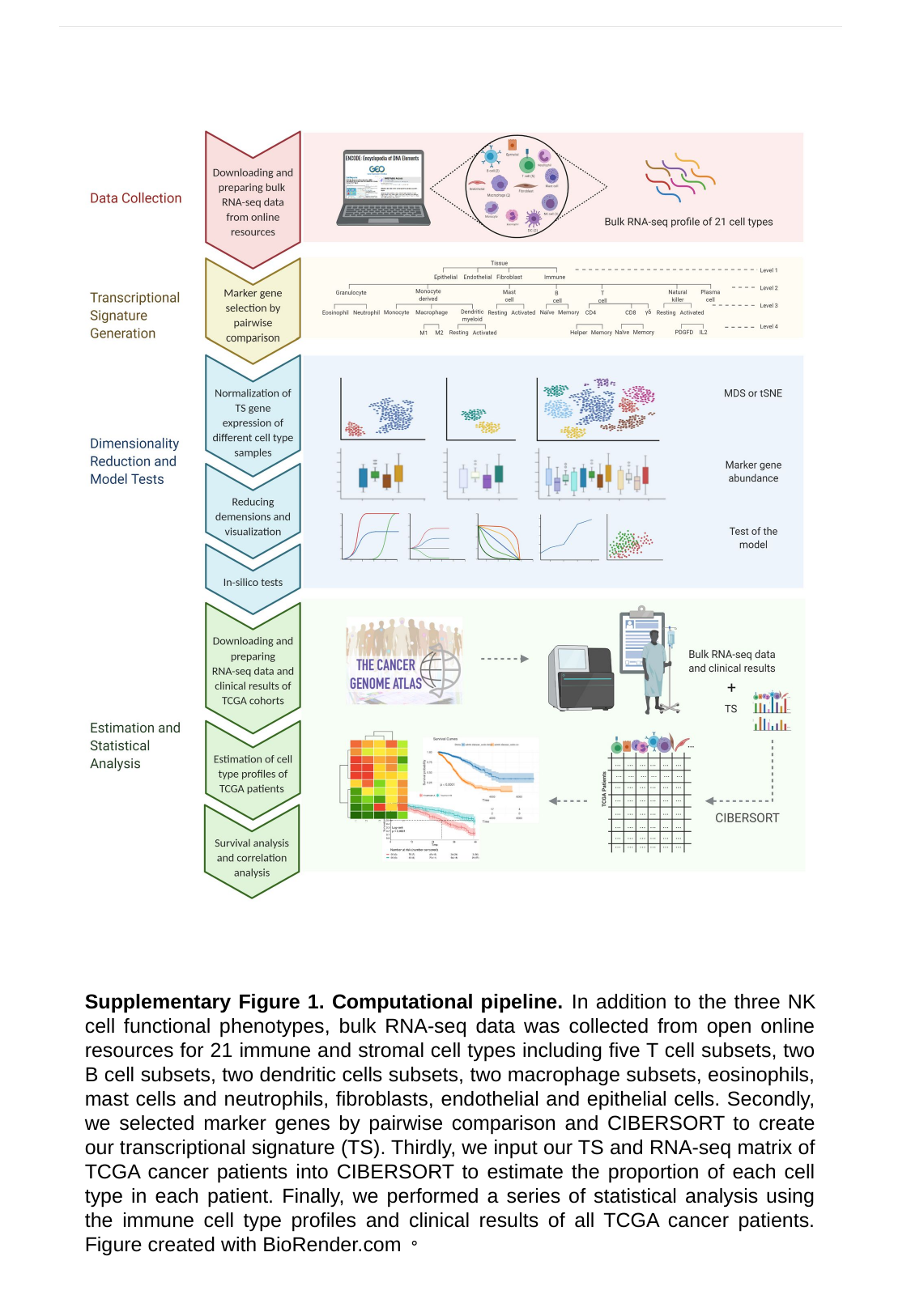

Supplementary Figure 1. Computational pipeline. In addition to the three NK cell functional phenotypes, bulk RNA-seq data was collected from open online resources for 21 immune and stromal cell types including five T cell subsets, two B cell subsets, two dendritic cells subsets, two macrophage subsets, eosinophils, mast cells and neutrophils, fibroblasts, endothelial and epithelial cells. Secondly, we selected marker genes by pairwise comparison and CIBERSORT to create our transcriptional signature (TS). Thirdly, we input our TS and RNA-seq matrix of TCGA cancer patients into CIBERSORT to estimate the proportion of each cell type in each patient. Finally, we performed a series of statistical analysis using the immune cell type profiles and clinical results of all TCGA cancer patients. Figure created with BioRender.com。

## Slide 2
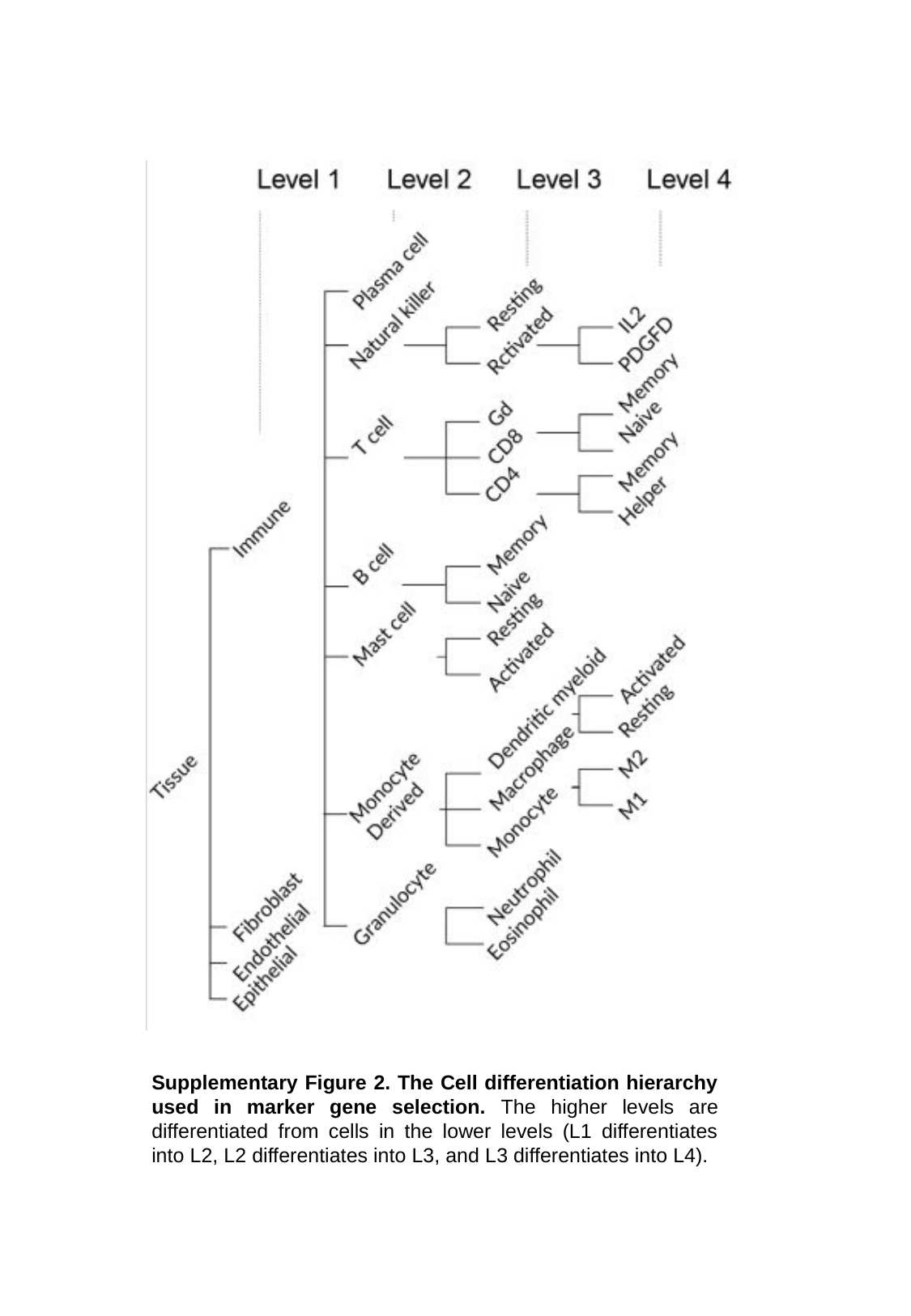

Supplementary Figure 2. The Cell differentiation hierarchy used in marker gene selection. The higher levels are differentiated from cells in the lower levels (L1 differentiates into L2, L2 differentiates into L3, and L3 differentiates into L4).

## Slide 3
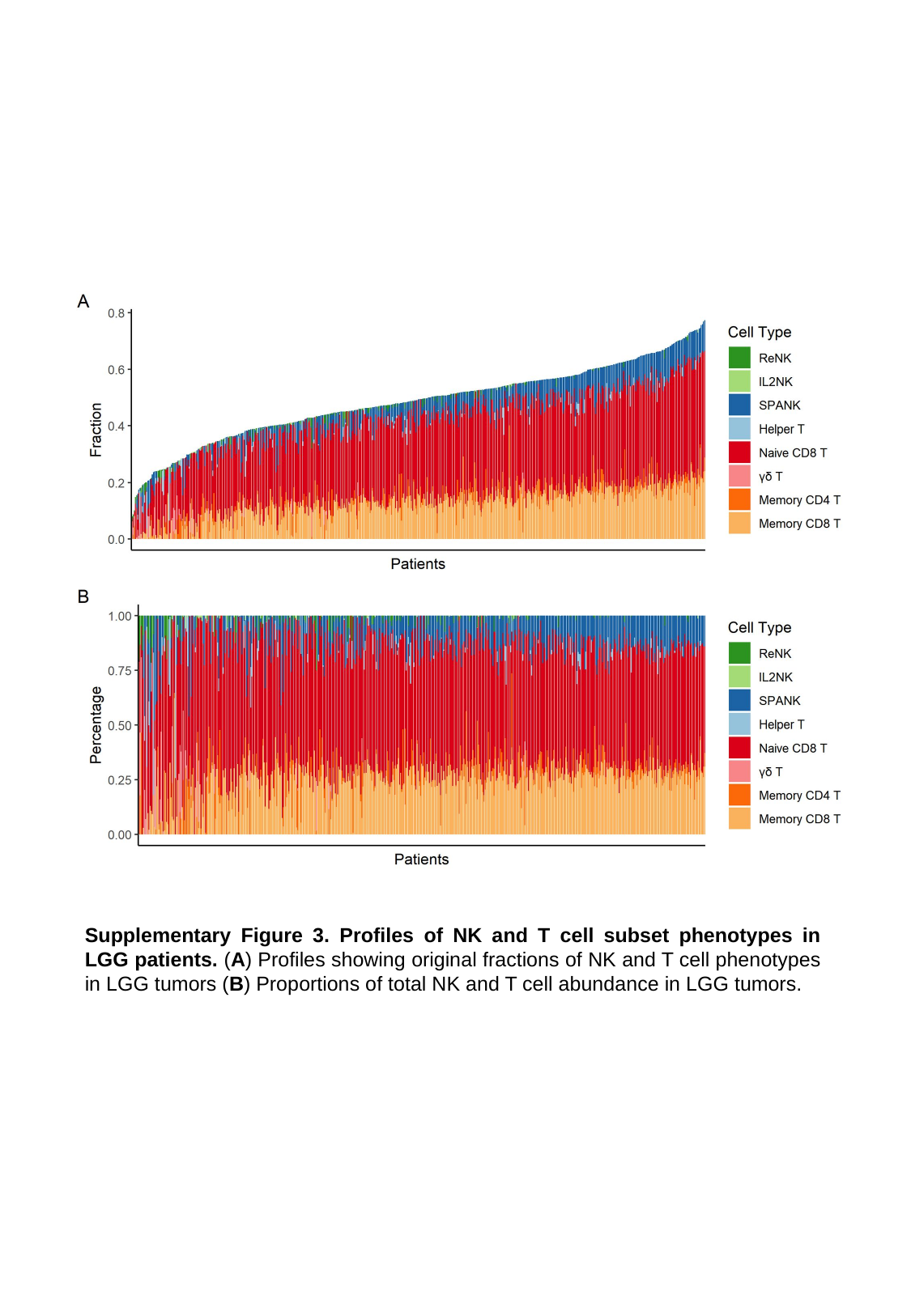

Supplementary Figure 3. Profiles of NK and T cell subset phenotypes in LGG patients. (A) Profiles showing original fractions of NK and T cell phenotypes in LGG tumors (B) Proportions of total NK and T cell abundance in LGG tumors.

## Slide 4
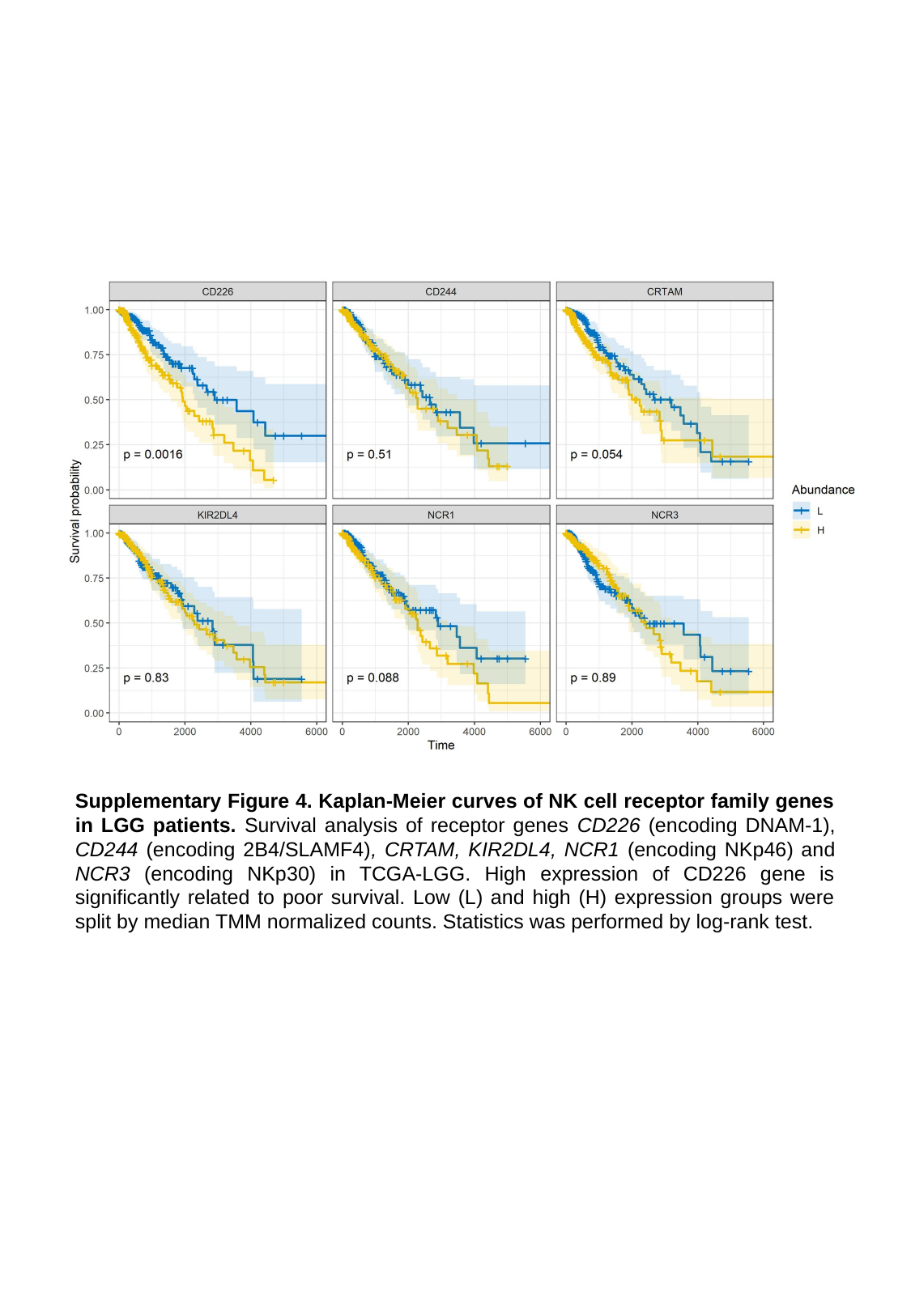

Supplementary Figure 4. Kaplan-Meier curves of NK cell receptor family genes in LGG patients. Survival analysis of receptor genes CD226 (encoding DNAM-1), CD244 (encoding 2B4/SLAMF4), CRTAM, KIR2DL4, NCR1 (encoding NKp46) and NCR3 (encoding NKp30) in TCGA-LGG. High expression of CD226 gene is significantly related to poor survival. Low (L) and high (H) expression groups were split by median TMM normalized counts. Statistics was performed by log-rank test.
